# Unilateral disruptions in the default network with aging in native space

**DOI:** 10.1002/brb3.202

**Published:** 2013-12-29

**Authors:** Qolamreza R Razlighi, Christian Habeck, Jason Steffener, Yunglin Gazes, Laura B Zahodne, Anna MacKay-Brandt, Yaakov Stern

**Affiliations:** Cognitive Neuroscience Division, Department of Neurology, Columbia University College of Physicians and SurgeonsNew York, New York

**Keywords:** Age-related brain change, cognitive performance, fMRI analysis, interhemispheric averaging, resting-state BOLD fMRI, spatial normalization, SPM

## Abstract

**Background:**

Disruption of the default-mode network (DMN) in healthy elders has been reported in many studies.

**Methods:**

In a group of 51 participants (25 young, 26 elder) we examined DMN connectivity in subjects' native space. In the native space method, subject-specific regional masks (obtained independently for each subject) are used to extract regional fMRI times series. This approach substitutes the spatial normalization and subsequent smoothing used in prevailing methods, affords more accurate spatial localization, and provides the power to examine connectivity separately in the two hemispheres instead of averaging regions across hemispheres.

**Results:**

The native space method yielded new findings which were not detectable by the prevailing methods. The most reliable and robust disruption in elders' DMN connectivity were found between supramarginal gyrus and superior-frontal cortex in the right hemisphere only. The mean correlation between these two regions in young participants was about 0.5, and dropped significantly to 0.04 in elders (*P* = 2.1 × 10^−5^). In addition, the magnitude of functional connectivity between these regions in the right hemisphere correlated with memory (*P* = 0.05) and general fluid ability (*P* = 0.01) in elder participants and with speed of processing in young participants (*P* = 0.008). These relationships were not observed in the left hemisphere.

**Conclusion:**

These findings suggest that analysis of DMN connectivity in subjects' native space can improve localization and power and that it is important to examine connectivity separately in each hemisphere.

## Introduction

The existence of coherent blood-oxygen-level-dependent (BOLD) signal in the lower frequencies among different brain regions at rest is commonly reported (Raichle 2009). The most well-known set of brain regions with coherent signal is referred to as the default-mode network (DMN) (Raichle 2011; Seibert and Brewer 2011). Age-related disruption in the coherence among these oscillating DMN brain regions has been reported in the absence of any disease (Andrews-Hanna et al. 2007). There have also been attempts to relate reduction in the strength of DMN functional connectivity with neurodegenerative diseases (Mevel et al. 2011; Wu et al. 2011; Seibert et al. 2012), and some have reported a relationship between the DMN connectivity and deposition of beta-amyloid (Persson and Nyberg 2006; Hedden et al. 2009). These observations increase interest in the study of age-related changes in the integrity of the DMN.

**Figure 1:**
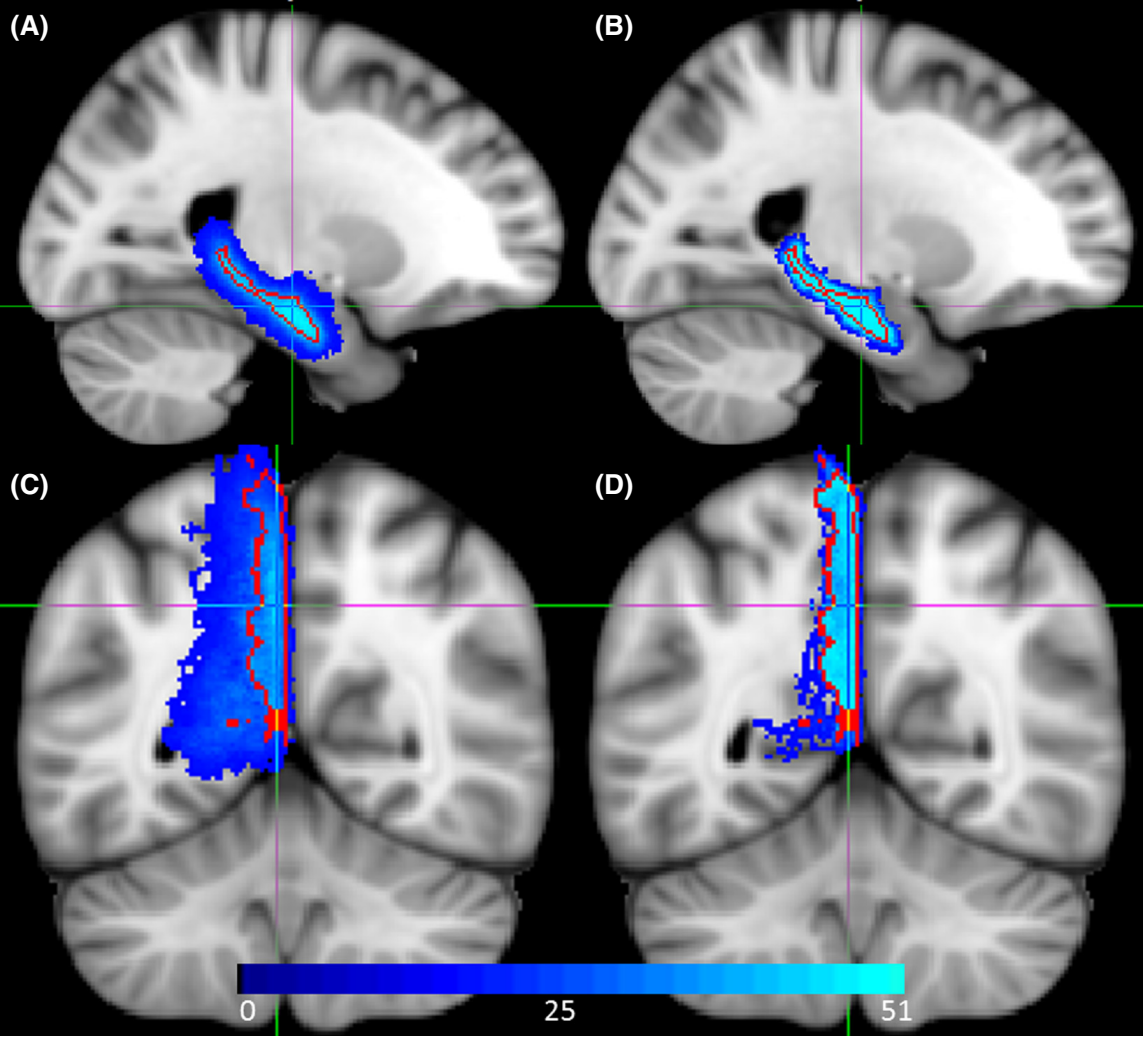
Color-coded overlay maps of (A) hippocampus and (C) precuneus regions on MNI152 brain atlas after statistical parametric mapping (SPM)8 spatial normalization. Color-coded overlay maps of (B) hippocampus and (D) precuneus regions on MNI152 brain atlas after region-based alignment. In red is the border of precuneus and hippocampus as defined in the atlas.

In this study, we investigated age-related changes in functional connectivity of the DMN by analyzing the resting-state BOLD fMRI data in subjects' native space instead of standardized atlas space. The native space approach substitutes for spatial normalization. Spatial normalization is the conventional method for warping all subjects in a study into a standard space and facilitates the use of predefine regions-of-interest (ROI) mask from the utilized brain atlas instead of the subjects own regional mask. It is implemented by registering each subject's brain to a canonical template brain. Spatial normalization is a key step for studies doing voxel-wise, across-subject comparisons. A long-standing problem in functional neuroimaging studies of aging is that the large age-related changes in brain morphology make it difficult to coregister brains (Yassa and Stark 2009; Seibert and Brewer 2011). Although spatial normalization is intended to fit all brain images to a standardized space, the assumption that any voxel represents the same brain location for every subject is typically untrue. To illustrate the extent of the problem caused by spatial normalization, we used FreeSurfer (http://surfer.nmr.mgh.harvard.edu/) to extract hippocampus and precuneus binary masks for the 51 subjects included in this study. We then used statistical parametric mapping (SPM8, Wellcome Department of Cognitive Neurology) to perform spatial normalization. The 51 spatially normalized masks were summed and overlaid on top of the MNI152 atlas. Figure 1A and C display these summed mask images, which we call an “*overlay map*” of the regions. The voxel values inside these *overlay maps* show how many subjects have their hippocampus/precuneus regions in that particular location. For instance, if the voxel value in the *overlay map* of the hippocampus is 51, then this voxel belongs to hippocampus in all the subjects after spatial normalization. Lower values indicate that fewer subjects have the same region in that voxel. As shown in Figure 1A and C, the *overlay maps* of the aforementioned two regions extend far beyond their borders (the red curve shows the border of two regions in the atlas) even after spatial normalization. This simple example clearly demonstrates the poor performance of the prevailing spatial normalization method for fMRI data analysis in aging research.

**Figure 2:**
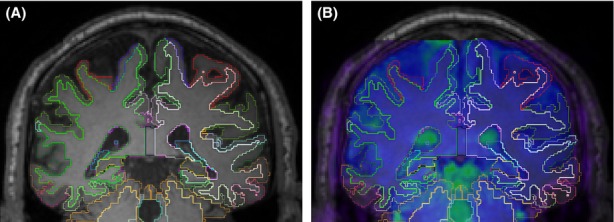
The typical atrophy in a healthy 63-year-old female participant's brain in a T1 scan, (A) FreeSurfer extracted cortical and subcortical ROI borders overlaid on the T1 scan; (B) FMRI reference image overlaid on (A) after intermodal registration using FLIRT. The dura-matter line and the ventriculars illustrate the accuracy of this registration. ROI, regions of interest; FLIRT, FMRIB's linear image registration tool.

It is common to apply a strong spatial smoothing on the fMRI data in order to compensate for the inaccuracy in spatial normalization. Even though smoothing reduces the rate of false positives, it also reduces the likelihood of detecting true positive. Nonetheless, in studies comparing young and old brains, even strong smoothing cannot compensate for the error introduced by spatial normalization due to the extent of the atrophied elders' brain. For instance, Figure 2A shows a 63-year-old healthy female participant's brain in our data set, illustrating atrophy exceeding the kernel size of any smoothing filter used in fMRI analysis. Another potential problem of spatial smoothing is that it makes it more difficult to segregate regions that are located close to each other. For instance, regions close to the middle hemispheric plane (i.e., left and right posterior cingulate) have to be treated as a single region. In fact, in the prevailing method of functional connectivity analysis with spatial normalization, it is a common practice to place the seed exactly on the middle plane and average all voxels' signal within a sphere centered by that seed. This subsequently forces interhemispheric averaging in the analysis of resting-state BOLD fMRI data. In addition, a recent study (Smith et al. 2011) showed that time series in atlas-based seed ROI's derived after spatial normalization and not from native space data are extremely damaging to the DMN estimations.

To address these issues, we analyzed fMRI data in subjects' native space, which substitutes the spatial normalization and subsequent smoothing. Analyzing fMRI data in subjects' native space requires a highly accurate method for reliably identifying neuroanatomical regions in fMRI image for every subject in the study, often referred to as fMRI localization (Gholipour et al. 2007). Direct fMRI localization is challenging as the overall brain structures are not clearly visible in fMRI scans. Instead, one can use the accompanying T1 image for the localization purpose and then transfer the localization data to the fMRI image. A structural T1 is typically acquired immediately before/after the functional fMRI data acquisition. In addition, the T1 image is often acquired in the same scanner and space of the fMRI data, which facilitate their intermodal coregistration. In this study, we took advantage of FreeSurfer's parcellation and segmentation (Fischl et al. 2002, 2004) to accurately locate ROI in the subject's native space. Then we transferred the FreeSurfer regional mask to fMRI space and obtained a single averaged resting-state BOLD signal in every region. This method enables us to compare regional connectivity in young and elder brains without requiring the problematic preprocessing steps of spatial normalization and smoothing. It also provides higher statistical power because location-specific signals are more accurately captured.

A similar method for analysis of resting-state BOLD fMRI data in surface space has been reported previously (Seibert and Brewer 2011). In that study, FreeSurfer was used to identify ROIs in native surface space on the cortex, whereas in the proposed method here we used the volumetric mask of each ROI (both for cortical and subcortical regions) to extract the regional signal.

We used the additional power afforded by this method to examine age-related changes in DMN connectivity in each hemisphere separately rather than the prevailing approach of averaging ROIs across hemispheres. Furthermore, we investigated whether this disruption is truly bilateral in nature or has unilateral characteristics. To investigate the effects of interhemispheric averaging, we repeated the native space analysis by averaging both hemispheres' regional time series in the analysis of resting-state BOLD fMRI data.

We compared the results of the proposed native space method to those obtained using the commonly adopted approach of spatial normalization and smoothing. Finally, in the DMN regions that are found to be significantly different between age groups, we examined the relationship between the strength of their functional connectivity and cognitive performance.

## Method

### Subjects

Twenty-five young healthy participants (11 M, 14 F, mean age: 25.36 years, SD age: 2.74 years), and 26-year-old healthy participants (12 M, 14 F, mean age: 65.11 years, SD age: 2.98 years) were recruited through random market mailing from within 10 miles of the Columbia University Medical Center. This recruitment approach is intended to obviate cohort effects that might be present by using convenience samples. All 51 subjects were right handed and did not differ regarding their level of education (young: 15.5 ± 2.06 years old: 15.27± 3.04 years). As can be seen, an extensive effort was made to make the two age groups comparable in their education, method of recruitment, geographical area of residence, male-to-female ratio, and within-group age distribution. Participants were screened to exclude individuals with a history of neurologic or psychiatric conditions and those using psychoactive medications. All subjects were compensated for participation. Informed consent was obtained prior to testing under supervision of the Columbia University Medical Center Institutional Review Board.

### Neuropsychological examination

A battery of neuropsychological tests was administered to all participants. Tests that putatively assess the following domains were selected; *memory*: three measures of immediate verbal memory from the selective reminding test (SRT; Buschke and Fuld 1974). *Speed of processing*: the digit symbol subtest from the Wechsler Adult Intelligence Scale–Version 3 (WAIS-3; Wechsler 1997), Trail Making Test A (Lezak et al. 2004), and the Stroop color naming condition (Golden 1975). *General fluid ability*: matrix reasoning, letter-number sequencing, and block design subtests from the WAIS-3. *Vocabulary*: the vocabulary subtest from the WAIS-3, Wechsler Test of Adult Reading (Wechsler 2001), and American National Adult Reading Test (Grober and Sliwinski 1991).

These Neuropsychological variables were reduced through confirmatory factor analysis (CFA) on a larger sample of 188 participants in neuroimaging studies in our laboratory. CFA was utilized to obtain the factor scores for the aforementioned cognitive domains. The a priori four-factor model of *memory*, *speed of processing*, *general fluid ability*, and *vocabulary* yielded acceptable fit statistics: root mean square error of approximation = 0.05, comparative fit index = 0.99; Tucker-Lewis index = 0.98. All indicator task loadings on their respective cognitive factors were at or above 0.68. Factor scores were outputted from Mplus Version 6.12 (Muthen and Muthen 1998).

### Data acquisition

Structural images were acquired using a 3.0 Tesla magnetic resonance scanner (Philips, Andover, MA). Structural image were obtained with T1-weighted turbo field echo (FE) high-resolution image with echo time (TE) = 2.98 msec; repetition time (TR) = 6.57 msec; flip angle = 8°; 256 × 256 matrix; in-plane voxel size = 1.0 × 1.0 mm; slice thickness = 1.0 mm (no gap); 165 slices.

Functional images were acquired using the same 3.0 Tesla magnetic resonance scanner with a FE echo planar imaging (FE-EPI) sequence (TE/TR = 20/2000 msec; flip angle = 72°; 112 × 112 matrix; in-plane voxel size = 2.0 × 2.0 mm; slice thickness = 3.0 mm [no gap]; 37 transverse slices per volume), 6:1 Philips interleaved, in ascending order. Participants were scanned for 9.5 min, with instructions to rest, to keep their eyes open for the duration of the scan, not to think of any one thing in particular, and not to fall asleep.

### MRI data reconstruction

Each subject's structural T1 scans were reconstructed using FreeSurfer (http://surfer.nmr.mgh.harvard.edu/). The accuracy of FreeSurfer's subcortical segmentation and cortical parcellation (Fischl et al. 2002, 2004) was reported to be comparable to manual labeling. Each subject's white and gray matter boundaries as well as gray matter and cerebral spinal fluid boundaries were visually inspected slice by slice, manual control points were added in the case of any visible discrepancy, and reconstruction was repeated until we reached satisfactory results within every subject. The subcortical structure borders were plotted by *freeview* visualization tools (part of FreeSurfer package) and compared against the brain regions. In case of discrepancy, they were corrected manually. A separate mask was generated for each and every segmented subcortical region and parcellated cortical region. These masks were transferred to the T1 native space using nearest-neighbor interpolation. The transformation matrix was obtained by registering the subject's head from FreeSurfer space to native space by FMRIB software library (FSL) linear registration tool (http://fsl.fmrib.ox.ac.uk/fsl/flirt/) with 6 degree of freedom (df), rigid-body, 256 bins normalized mutual information cost function, and trilinear interpolation. Quality check was performed by overlaying the masks on top of the T1 image in the subject's native space. No discrepancy was found at this stage.

### Resting BOLD fMRI preprocessing

The 6:1 slice interleaving of Philips scanner was corrected using *Sinc* interpolation using SPM8 software package. Our MRI protocol did not include bias field map acquisition, thus we could not correct for B0 field inhomogeneity correction. However, correlations in temporal signals are not altered with the mean of the signals, therefore the effect of B0 field inhomogeneity in the absence of spatial smoothing is negligible. It should be emphasized that spatial smoothing is not required in fMRI data analysis in native space. However, this does not rule out the effect of B0 field inhomogeneity in the intermodal registration of fMRI and T1 scans. This will be discussed next in the next section.

**Figure 3:**
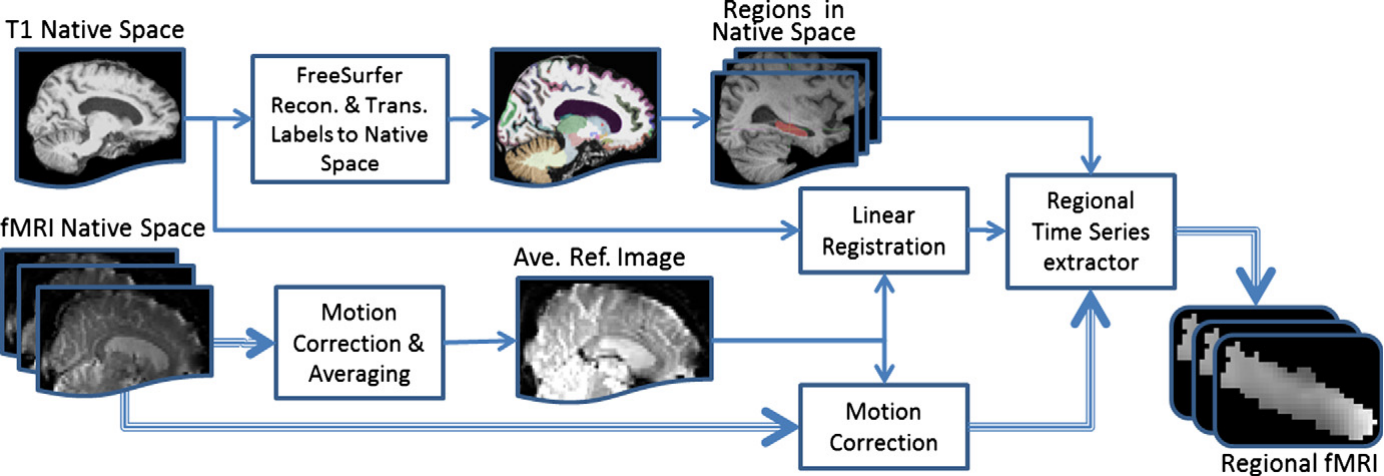
Flowchart of the fMRI data analysis in subject's native space. The thick triple line shows the flow of the fMRI data.

There have been many reports of motion-induced correlation between ROIs in resting-state BOLD fMRI data (Birn et al. 2006; Power et al. 2012; Van Dijk et al. 2012; Carp 2013), so extra caution was taken in this study to deal with this issue (see Fig. 3). We used *mcflirt* (motion correction tools in the FSL package [Jenkinson et al. 2012]) to register all the volumes to a reference image (Jenkinson et al. 2002). The reference image was generated by registering (6 df, 256 bins mutual information, and *Sinc* interpolation) all volumes to the middle volume and averaging them. We made sure that the selected middle volume was free of artifacts and motion by examining the derivative of the transformation parameters around that volume. We then used the method described in Power et al. (2012) to calculate frame-wise displacement (FD) from the six motion parameters and root mean square difference (RMSD) of the bold percentage signal in the consecutive volumes for every subject. To be more conservative, we lowered the threshold of our RMSD to 0.3%. (It was originally suggested to be 0.5%.) RMSD was computed on the motion-corrected volumes before temporal filtering. The contaminated volumes were detected by the criteria FD > 0.5 mm or RMSD > 0.3%. Identified contaminated volumes were replaced with new volumes generated by linear interpolation of adjacent volumes. Volume replacement was done before band-pass filtering (Carp 2013).

The motion-corrected signals were passed through a band-pass filter with the cut-off frequencies of 0.01 and 0.08 Hz. This band-pass filter has three functions: First, it is an antialiasing filter to remove aliasing due to 0.5 Hz sampling of the BOLD signal; second, it eliminates the higher frequency (>0.1 Hz) fluctuations of the BOLD signal that are mainly a reflection of respiration signal modulated by heartbeat signal; third, it removes the high-power low-frequency noise (the power spectrum of the noise is related to the frequency by 1/f factor). We used *flsmaths–bptf* to do the filtering in this study (Jenkinson et al. 2012). After filtering, the first few volumes were discarded due to the lag of the digital filter. Anecdotal observations in our division showed that digital filter lags (almost the same as the order of the filter) often induce minor correlations between the signals.

Finally, we residualized the motion-corrected, scrubbed, and temporally filtered volumes by regressing out the FD, RMSD, left and right hemisphere white matter, and lateral ventricular signals (Birn et al. 2006). We expected that volume scrubbing would effectively remove sudden but large movements of the head and that subsequent residualization would further remove the effect of steady but small motion of the head often found in older subjects due to respiration or tremor.

### FMRI analysis in native space

Figure 3 presents the flowchart of the processes in our native space method. T1 image segmentation and parcellation were done by FreeSurfer. The FreeSurfer segmentation and parcellation results were then transferred to the subject's native space. A separate mask was generated for every segmented subcortical and parcellated cortical region for each subject.

Intermodal, intrasubject, rigid-body registration of fMRI reference image and T1 scan is a challenging task. We examined three intermodal registration methods, FMRIB's linear image registration tool (FLIRT) (Jenkinson et al. 2012), boundary-based registration (BBR) (Greve and Fischl 2009), and advanced normalization tools (ANTS) (Avants et al. 2011), for 10 randomly selected subjects in our data set. Visual inspection showed that the results of FLIRT and BBR algorithms are very similar and outperform ANTS. Even though BBR algorithm claims to be robust to B0 field inhomogeneity (Greve and Fischl 2009), FLIRT performance was slightly better than BBR in registering the two modalities. We used the same reference image generated for motion correction to register the structural T1 image to fMRI space using FLIRT with 6 df, 256 bins normalized mutual information, and trilinear interpolation (Jenkinson and Smith 2001). The results of this intermodal registration were examined visually for all 51 subjects in our data set using *Freeview* visualization tools, overlaying fMRI reference image, and delineated T1 scan. Figure 2B illustrates a sample result of our intermodal registration. As can be seen in the figure, FreeSurfer's extracted region's borders facilitate this visual inspection. This time-consuming process of visual inspection also examined the effect of EPI spatial distortion and B0 field inhomogeneity after intermodal registration. Even though this visual inspection did not reveal any major intermodal registration inaccuracy, it was a crucial step in our project as our data do not include the reverse polarity acquisition which is often used for spatial distortion correction. Using the computed transformation matrix and FreeSurfer's generated masks in the subject's native space, the regional fMRI data were extracted from each subject's data. At this stage, the extracted regional fMRI data are in each subject's native space and stored separately for each subject and ROI. Only one interpolation was used in the entire process of localization by combining the transformation parameters for all three realignments: (1) motion correction, (2) FreeSurfer to T1, and (3) T1 to averaged reference image in fMRI space. This minimizes the effect of nearest-neighbor interpolation errors in the final outcome.

Ten regions of DMN were considered in this study and have been repeatedly reported in the literature (Andrews-Hanna et al. 2007; Buckner and Vincent 2007; Buckner et al. 2008; Raichle 2011). The names of the neuroanatomical regions in DMN and their abbreviations are as follows: hippocampus (Hi), entorhinal cortex (En), inferior parietal lobule (IP), isthmus of the cingulate (IC), medial orbitofrontal cortex (MOF), parahippocampal gyrus (PHi), posterior cingulate (PoC), precuneus (PCu), superior-frontal gyrus (SF), and supramarginal gyrus (SM). Once the ten regional fMRI images were extracted separately for each subject, temporal BOLD signal was calculated for each region by averaging all voxels inside the region.

For comparison purpose, we complemented the native space analysis with the prevailing spatial normalization and smoothing in SPM8 software package, whereas the rest of the processing pipeline remained the same. We used the MNI152 as the standard template and smoothing was done by a Gaussian kernel of full width at half maximum (FWHM) equal to 6 mm. The same DMN region masks in MNI152 template space were used to extract the 10 regional time series for every subject after spatial normalization and smoothing.

To examine the effect of averaging the left and right hemispheres (Vincent et al. 2006; Andrews-Hanna et al. 2007; Buckner et al. 2008; Hedden et al. 2009), analyses were repeated after averaging the left and right temporal signal for every corresponding neuroanatomical region.

### Statistical analysis

Once the averaged signal of each region was obtained, Fisher *Z*-transformed correlation coefficients were computed for each possible pair of neuroanatomical regions included in the DMN. These analyses were done separately for each hemisphere. In total, we computed 2×(10 × 9/2) = 90 pair-wise interregional correlation coefficients for each subject. The group mean was computed for each interregional pair, and two-sample *T*-tests were performed to detect age group mean differences in interregional functional connectivity. Significant differences between the young and elder groups' DMN functional connectivity were determined before (*P* < 0.05) and after Bonferroni correction (*P* < 0.05/90). To investigate the unilateral age effect on brain hemispheres, a regression analysis was carried out with age, hemisphere, and their interaction term as independent variables, and the functional connectivity as the dependent variable.

### Correlation with cognition

Linear regression was used to examine the relationships between the cognitive factor scores and the magnitude of functional connectivity, focusing on the DMN regions where connectivity was significantly different across the age groups. This linear model was independently fitted for young and elder groups to investigate this relationship separately in each group. We also added age as an independent variable in our linear model to remove any possible within-group age effect.

## Results

**Figure 4:**
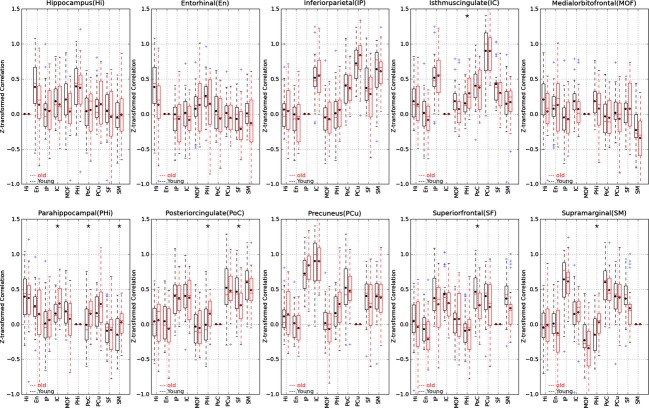
Pair-wise Fisher *Z*-transformed correlations of the default network regions in boxplot format for left hemispheres. The box extends from the lower to upper quartile values of the data, with a line at the median. The whiskers extend from the box to show the range of the data when the outlier points are excluded. Outlier points are those that fall outside 1.5 times the interquartile range (0.25–0.75 quartile). The black dots show the means of the groups, the single asterisk is the indicative of significance difference with 0.00056 < *P* < 0.05, and the double asterisks is the indicative of significance level after Bonferroni correction *P* < 0.00056.

**Figure 5:**
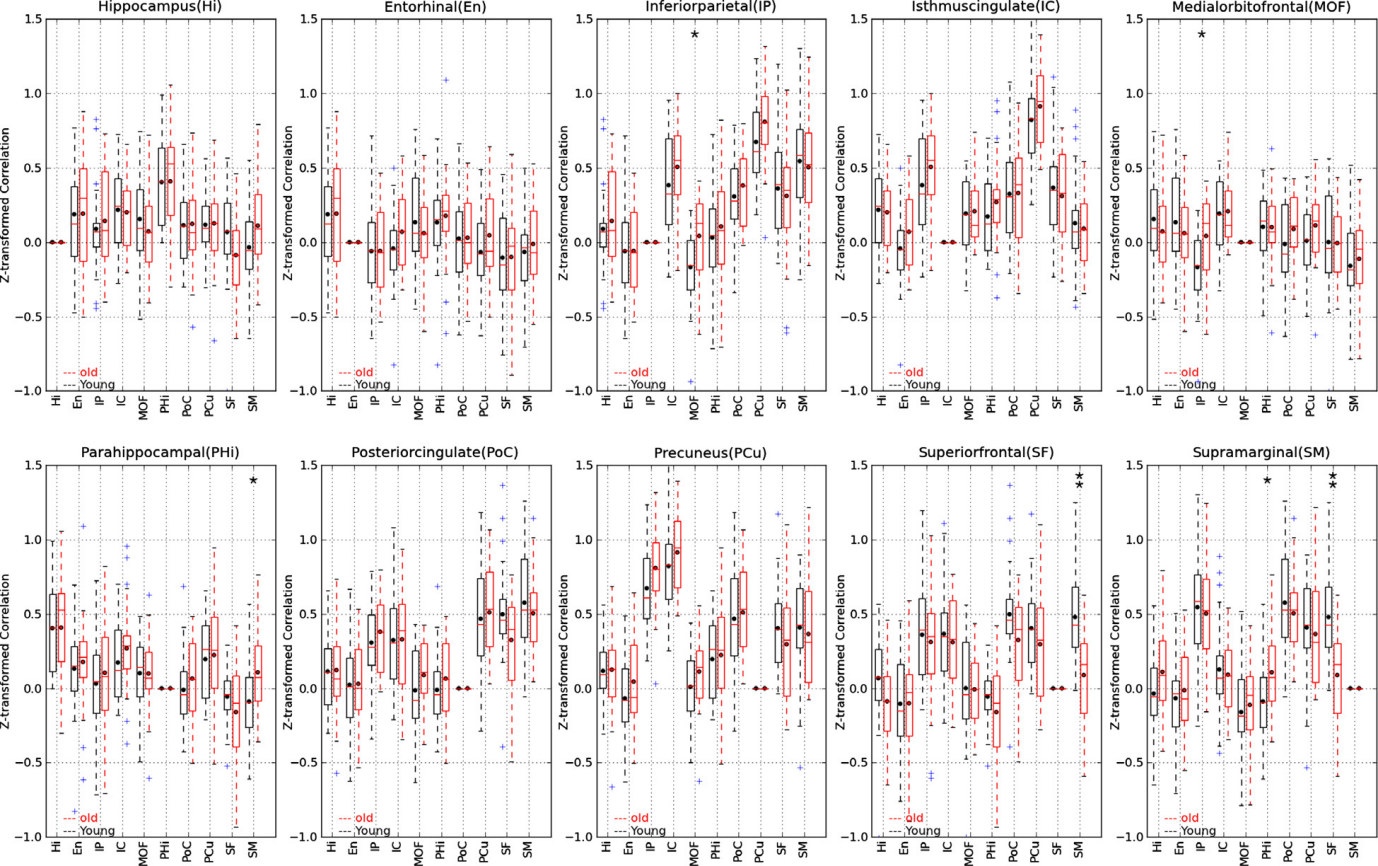
Pair-wise *Z*-transformed correlations of the default network regions in boxplot format for right hemispheres. The box extends from the lower to upper quartile values of the data, with a line at the median. The whiskers extend from the box to show the range of the data when the outlier points are excluded. Outlier points are those that fall outside 1.5 times the interquartile range (0.25–0.75 quartile). The black dots show the means of the groups, the single asterisk is the indicative of significance difference with 0.00056 < *P* < 0.05, and the double asterisks is the indicative of significance level after Bonferroni correction *P* < 0.00056.

**Figure 6:**
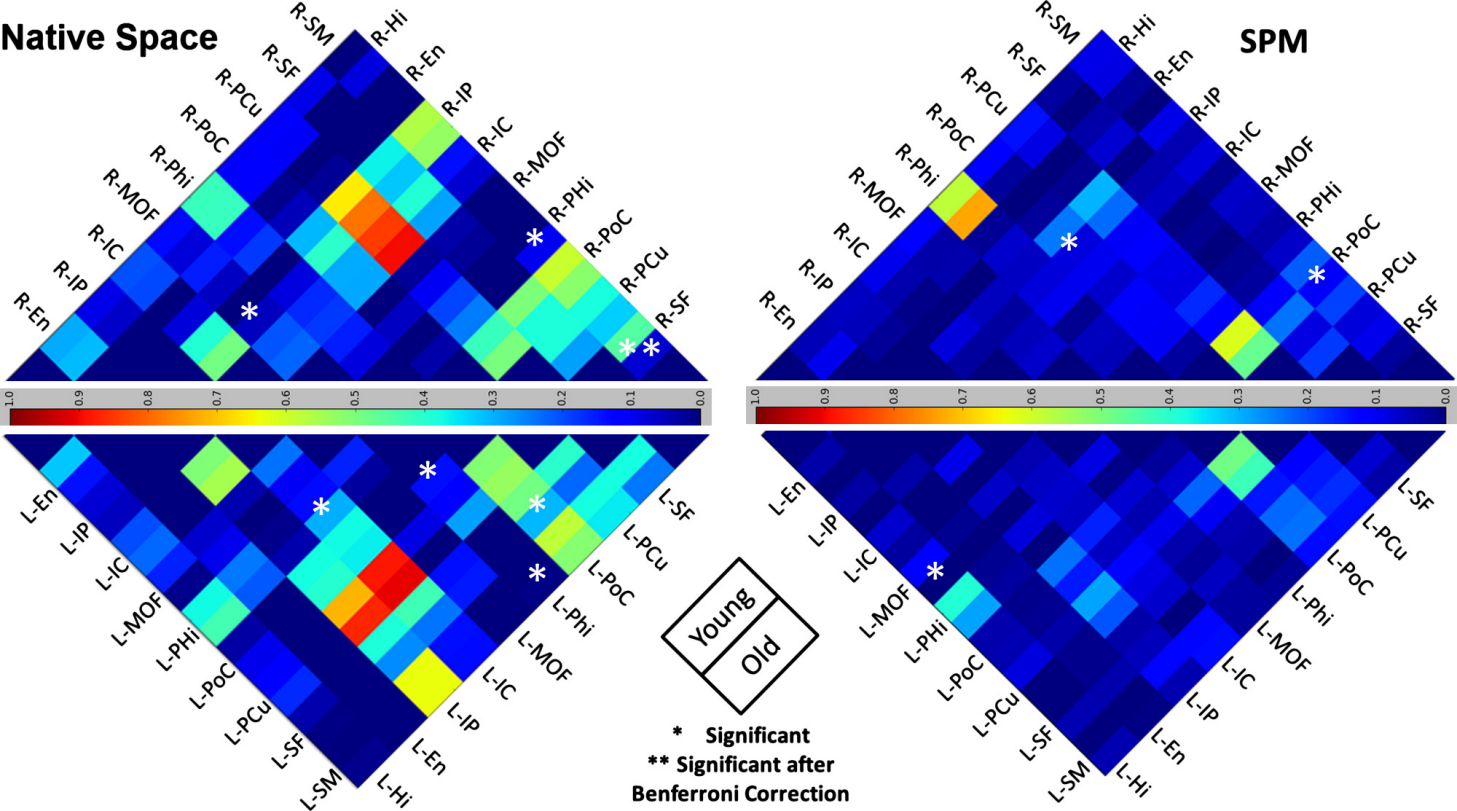
Color-coded cross-correlograms for correlation means of 10 FreeSurfer extracted ROIs for 51 subjects in study. Significant age-related disruptions in default network are marked by asterisks. Right/left hemisphere correlations means are in the upper/lower triangular. Left: results from our native space technique that uses subject-specific ROI extractions via FreeSurfer; right: results using traditional SPM8 voxel-wise coregistration and spatial normalization. ROI, regions of interest; SPM, statistical parametric mapping.

Figure 1 demonstrates a qualitative assessment of the localization accuracy achieved by native space method. Although prevailing method of spatial normalization in SPM8 extends the *overlay maps* of the hippocampus and precuneus regions far beyond their border (Fig. 1A and C), the native space method constrains the *overlay maps* to the border of the two regions (Fig. 1B and D). Figures 4 and 5 illustrate the pair-wise Fisher *Z*-transformed correlations of the DMN regions in boxplot format for left and right hemispheres, respectively. Each subplot in Figures 4 and 5 shows the intrahemispheric correlations of each neuroanatomical region with the remaining nine regions in DMN. The title of each subplot gives the neuroanatomical region name. In these boxplots, the box extends from the lower to upper quartile values of the data, with a line at the median. The whiskers extend from the box to show the range of the data when the outlier points are excluded. Outlier points are those that fall outside 1.5 times the interquartile range (0.25–0.75 quartile). The black dots show the means of the groups, the single asterisk is indicative of significant difference with 0.00056 < *P* < 0.05, and the double asterisks is the indicative of significance level after Bonferroni correction *P* < 0.00056. Figure 6 summarizes both Figures 4 and 5 into a single cross-correlogram. The right and left hemisphere DMN interregional correlation means are shown in upper and lower triangles, respectively. Each diamond is divided into two parts, which display the mean correlation of young and elder groups for its two crossing regions. Again, double asterisks indicate significant differences that survive the Bonferroni correction (*P* < 0.00056), whereas single asterisks indicate significant differences in an uncorrected *T*-test (0.00056 < *P* < 0.05) that do not survive the Bonferroni correction. Figure 6 makes it easier to see the differences in the group mean values through a color-coded graph. There are seven interregional correlations in the DMN that showed a significant difference between young and elder groups: four in the left hemisphere; (PHi, SM), (PHi, PoC), (PHi, IC), and (PoC, SF), and three in the right hemisphere; (PHi, SM), (IP, MOF), and (SM, SF). However, only the age-related difference in functional connectivity between SM and SF in the right hemisphere remained significant after Bonferroni correction (*P* = 0.000021). Five of the differences reflect an increase in functional connectivity in elders, whereas two pairs (one in right hemisphere; [SM, SF], and one in left hemisphere; [PoC, SF]) show a decrease in the functional connectivity in elders. Only one interregion (PHi, SM) connectivity was significantly different bilaterally (in both hemispheres), whereas the rest of the findings are unilateral (i.e., are found only in one hemisphere) including the one significant finding that survived Bonferroni correction.

A regression analysis investigating the correlation between SM and SF indicated significant hemisphere (*P* = 0.04) and Age × Hemisphere interaction terms (*P* = 0.03). This indicates a significant difference between age effect on connectivity in the two hemispheres.

### Comparison with SPM8

For comparison with the native space method, we also calculated mean correlation between nodes of the DMN for young and elders after processing using the prevailing method of fMRI analysis performed by SMP8. All aspects of the data analysis for these two processes were identical; only the SPM8 spatial normalization and smoothing was replaced with native space analysis in our study. The results of the prevailing method by SPM8 are also summarized in Figure 6, which presents a color-coded cross-correlogram. It is clear from Figure 6 that the overall mean correlation between the DMN nodes was higher when using the native space method.

Using SPM8-based spatial normalization, we found three interregional correlations in the DMN that were significantly different between young and elder groups: one in the left hemisphere (Hi, MOF); and two in the right hemisphere; (PoC, SM) and (PoC, IP). However, none of correlations survived Bonferroni correction (*P* < 0.00056). All three differences were unilateral and reflected a decrease in functional connectivity in elders. Most interestingly, none of the findings with SPM8 spatial normalization coincides with the findings in native space. However, the significant decrease noted by this method in the left hemisphere between (Hi, MOF) was also marginally significant in native space analysis (*P* = 0.06).

### Interhemispheric averaging

**Figure 7:**
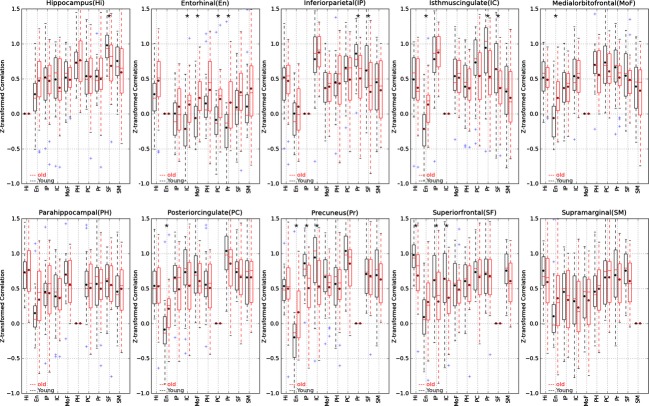
Pair-wise *Z*-transformed correlations of the default network regions in boxplot format for averaged right and left hemispheres. The box extends from the lower to upper quartile values of the data, with a line at the median. The whiskers extend from the box to show the range of the data when the outlier points are excluded. Outlier points are those that fall outside 1.5 times the interquartile range (0.25–0.75 quartile). The black dots show the means of the groups, the single asterisk is the indicative of significance difference with 0.00056 < *P* < 0.05, and the double asterisks is the indicative of significance level after Bonferroni correction *P* < 0.00056.

**Figure 8:**
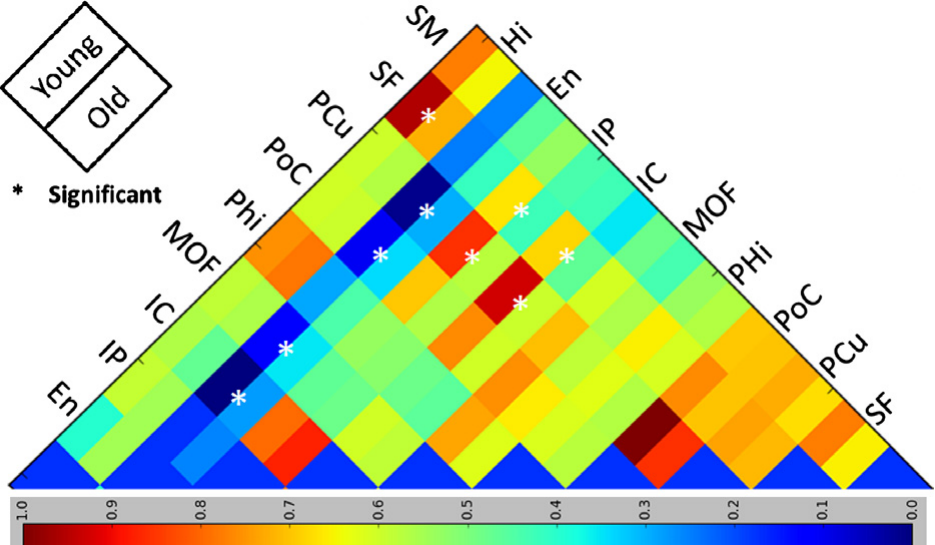
Right/left hemisphere averaged cross-correlograms for correlation mean of default network regions for 51 subjects in the study. Significant disruptions in default network are marked by asterisks.

Figure 7 illustrates the effect of interhemispheric averaging on the detection of age-related differences in the resting-state BOLD fMRI regional activation. As seen in this figure, interhemispheric averaging has multiple effects on the correlation statistics. This is better illustrated in the cross-correlogram in Figure 8. Comparing Figures 6 and 8 clearly shows that interhemispheric averaging increases the overall correlation mean significantly in both groups. However, there exist a few cases where averaging reduces the mean correlation (e.g., IP and SM). In general, when the mean correlations in the left or the right hemispheres are close to zero, averaging tends to inflate those correlations. One can roughly observe the following relationship: the higher the interhemispheric correlation, the smaller the effect of the averaging. Nine significant (*P* < 0.05) age-related changes were detected in the averaged signals: (En, PCu), (En, PoC), (En, MOF), (En, IC), (Hi, SF), (IP, SF), (IP, PCu), (IC, SF), and (IC, PCu). None survive after Bonferroni correction. Among these findings, five pairs showed a decrease in functional connectivity and four showed an increase in functional connectivity in elders. None of the nine detected significant changes was found without averaging left and right signals. However, two of them ([Hi, SF]; *P* = 0.07, and [IP, PCu]; *P* = 0.08) were marginally significant in the right hemisphere. The remaining seven significant findings were not found in left or right hemisphere using native space method or the prevailing method by SPM8 in which interhemispheric averaging was not performed.

The results of the native space method showed a single pair of regions (Phi, SM) whose functional connectivity significantly increased with age in both hemispheres. After interhemispheric averaging, however, this age-dependent change in functional connectivity was not found. A more important consequence was that the highly significant age-related difference in connectivity between supramarginal and superior-frontal regions, which was detected only in the right hemisphere prior to averaging, was lost by averaging the signals.

### Correlation with cognition

**Figure 9:**
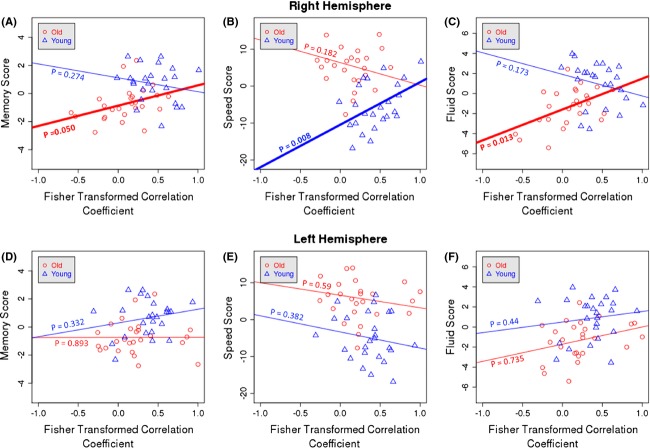
Relationship between right and left hemisphere functional connectivity between supramarginal gyrus and superior-frontal cortex on the right (A–C) and left (D–F) hemisphere and cognitive domains' factor scores: memory (A and D), speed of processing (B and E), general fluid ability (C and F).

Using linear regression models, we examined the relationship between functional connectivity and cognition across the seven regional pairs that were found to be altered significantly by age. These analyses were performed separately in the young and elder groups. Connectivity in only one of the seven region pairs with significant age-related DMN functional connectivity disruption (supramarginal and superior-frontal on the right hemisphere) was correlated with cognitive performance; connectivity in the remaining six significant findings was not found to be related to any of the cognitive domains' factor scores in the young or old subject groups. It is interesting to note that the age-related disruption in functional connectivity between SM and SF in the right hemisphere was also the only finding that survived Bonferroni correction (*P* < 0.00056). In the elder participants, the magnitude of functional connectivity of the SM and SF in the right hemisphere was correlated with better *memory* (*P* = 0.050) and *general fluid ability* (*P* = 0.013), but not with *speed of processing* (*P* = 0.182) or *vocabulary* (*P* = 0.192). In young participants, the magnitude of functional connectivity between SM and SF regions in right hemisphere was related to better *speed of processing* (*P* = 0.008), but not to *memory* (*P* = 0.274), *general fluid ability* (*P* = 0.173), or *vocabulary* (*P* = 0.772). Functional connectivity between the same regions in the left hemisphere was not related to cognition in either age group. Results of the correlation between SM and SF functional connectivity and cognitive performance are summarized in Figure 9. We excluded *vocabulary* for this figure as we did not find it to be correlated with any of our significant connectivity findings.

## Discussion

In this study, we explored age-related disruption in the functional connectivity among 10 neuroanatomical regions consistently reported as part of the DMN (Buckner et al. 2008; Raichle 2011; Seibert and Brewer 2011). The main goals of this study were to detect any differences in the functional connectivity of the DMN by age group using a new native space approach, explore hemisphere-specific connectivity, and relate DMN regions with age-dependent functional connectivity change to cognitive performance. In studies comparing young and elders, the already challenging issue of spatial normalization becomes even more problematic due to the atrophied brains in older adults, rendering the nonlinear registration step more difficult. To address this issue, we substituted the nonlinear registration and smoothing steps by analyzing the fMRI data in the subjects' native space. We used the structural T1 image acquired at the same time as the fMRI data to perform the localization by reconstructing the T1 image through FreeSurfer. FreeSurfer's regional masks were then used to extract the regional fMRI image from the whole-brain fMRI data. Spatial averaging of the fMRI data was performed within each region to get a single time series (signal) for each region. This approach increases power because it ensures that brain regions under examination are identical for each subject. The spatial averaging of voxels' data within a region should not be considered equivalent to spatial smoothing. Spatial smoothing is a weighted averaging of an area in the size of the smoothing filter's kernel (FWHM = 6–8 mm) regardless of the spatial location. Spatial smoothing thus often blends the signals from different tissue types (white matter, gray matter, and cerebro-spinal fluid) which has a drastically damaging effect on results as is demonstrated in a recent study (Smith et al. 2011). However, spatial averaging within a region in the proposed native space method only combines the signals from voxels that are carefully delineated and are supposed to belong to only one tissue type.

We demonstrated that the native space method was able to detect age-related differences in the integrity of the DMN across regions reported in studies that used the prevailing spatial coregistration method on data from larger groups of subjects (Andrews-Hanna et al. 2007). Previous studies using standard analytic techniques with sample sizes comparable to this study could not detect these differences (Bluhm et al. 2008; Beason-Held et al. 2009; Koch et al. 2010).

A similar method was proposed by Seibert and Brewer (2011) which is based on the native surface of the brain cortex rather than native volumes. In the native surface method, the vertices in the center of the gray matter of the cortex were considered as seed points, whereas in this work we averaged all voxels inside the ROI to obtain the regional time series. One advantage of our proposed method is that it is easily extendable to subcortical regions, whereas for native surface method this becomes challenging. However, a comprehensive comparison of the two methods is necessary to be able to thoroughly evaluate the relative effectiveness of the two methods.

As shown in Figures 6, we found seven significant age-related differences in the functional connectivity of DMN regions. After Bonferroni correction, only one significant difference remained: Connectivity between SM and SF regions in the right hemisphere. Interestingly, this age-related difference in connectivity was not noted in the left hemisphere. This may suggest that the disruption in the default network by age might be more of a unilateral process than a bilateral one. Some task-based fMRI studies have already reported hemispheric asymmetry alteration of brain activity by age during the task performance (Cabeza 2002), and resting-state cerebral blood flow (Lu et al. 2011). However, to the best of our knowledge it has not been reported for DMN using resting-state BOLD fMRI data.

We also compared our results with those obtained using the prevailing method in SMP8, which involves the typical spatial normalization by coregistering to MNI152 template and utilizing a set of predefined regional mask as the ROI across all the subjects in the study. However, utilization of data-driven atlases has gained popularity in recent years. They generate group-specific templates, and then a single standard space is derived from those templates. Spatial normalization in this case is done in two steps, first nonlinear registration to group-specific template and then to the standard space template. This can certainly improve the accuracy of the nonlinear registration. However, utilization of highly accurate nonlinear registration for spatial normalization is hampered by overfitting problem. That is why most of the existing software packages (SPM, analysis of functional neuroimages, FSL, etc.) use a mild or moderate level of nonlinear registration in their spatial normalization. In either case, comparing the effectiveness of our approach to normalization with different atlas (data-driven or standard) is beyond the scope of this study, as our native space method totally eliminates the need for spatial normalization.

The standard method produced three significant findings that did not survive Bonferroni correction and did not agree with any of the findings obtained with the new native space method. Only the change between one region pair (Hi, MOF) was found to be marginally significant in the same hemisphere (left) in the native space analysis. Figure 6 also shows that spatial smoothing reduces the overall mean of the pair-wise correlations between the DMN nodes. The fact that we did not detect any significant changes after Bonferroni correction in elders' DMN functional connectivity using SPM8 should not be surprising as many existing studies of age-related change in DMN have also failed to detect this difference (Bluhm et al. 2008; Beason-Held et al. 2009; Koch et al. 2010). Erroneous spatial normalization accompanied by strong spatial smoothing can simply cause a blending effect across regions which can deteriorate the contrast of the interregional functional connectivity between two groups.

There are growing numbers of studies that consider the decline in functional connectivity in DMN as biomarker/hallmark of age-related cognitive decline. However, as it is acknowledged in Hafkemeijer et al. (2012), it is very much possible that the age-related decline in the functional connectivity of the elders' DMN could be due to their significant brain atrophy. This is the issue addressed by our native space method. In the native space method, only gray matter voxels are considered in the analysis. These voxels are detected for each subject independently. That is why there is no blending of tissue types or spatial smoothing involved in this method. None of the existing work detects voxel location with such great accuracy. Another study (Damoiseaux et al. 2008) attempted to account for between-age-group morphological variations by adding the averaged gray-matter volume of all the default network regions as an independent variable in their statistical analysis. The problem associated with this approach is that the variation in the subjects' brain size even within groups is significantly high. This issue is often addressed by normalizing the gray-matter volume with intracranial volume. However, Damoiseaux et al. (2008) dealt with this problem by affine transferring the subjects' brains into a standard space. In other words, the subjects' brain volumes were increased/decreased to match to the size of the standard brain (which possibly removed the effect of atrophy) and then the averaged gray matter was computed. This would be much more compelling if it is done in native space.

It has been common practice to average the left and right hemispheres' resting-state BOLD fMRI data to achieve higher statistical power in the correlation values (Vincent et al. 2006; Andrews-Hanna et al. 2007). We directly examined the effect of interhemispheric averaging. We averaged the corresponding regional time series in left and right hemispheres in our data and reported the results in Figures 7 and 8. Interhemispheric averaging produced nine interregional pairs in DMN, whose functional connectivity differed significantly by age, but none of these findings survived Bonferroni correction. These observed significant findings were not detected in the individual hemispheres by both the native space method and the prevailing method by SMP8. Importantly, the significant age-related change in functional connectivity between SF and SM in the right hemisphere was lost by interhemispheric averaging. In cases where mean functional connectivity is small, interhemispheric averaging tended to increase the functional connectivity. However, there are also some regions (e.g., IP and SM) for which measured functional connectivity was reduced by interhemispheric averaging. These results suggest that interhemispheric averaging has a mixed effect (Razlighi et al. 2013). Our findings also suggest that the disruption in the DMN is distinct for each hemisphere, and averaging across hemispheres may obscure important information.

We also examined the relationship between the magnitude of functional connectivity and cognitive performance on four factors (*memory*, *speed of processing*, *general fluid ability*, and *vocabulary*) in regions where age-related differences in connectivity were noted. Connectivity between SM and SF was correlated with cognition in both groups; however, the cognitive domains that correlated with the magnitude of functional connectivity in that region differed for the young and elder groups. Although the nature of the relationship between functional connectivity and cognition in this age-sensitive region requires further study to fully understand the associations, the correlation with cognition suggests that connectivity between these two regions may have functional significance.

Beside DMN, there are other resting-state networks that are reported in the literature (Raichle 2011) such as dorsal attention network, executive control network, etc. We also used our native space method to examine any age-related changes in the pair-wise functional connectivities between main nodes of these networks. However, none of the findings survived Bonferroni correction.

Another important consideration in measuring functional connectivity with Pearson correlation coefficients is effect size. This has often been ignored in the literature. As we are quantifying functional connectivity by computing the Pearson correlation coefficient between two BOLD signals with 285 times points, a simple *T*-test might not be sufficient to make a meaningful conclusion on data with very small correlation (<0.2). The effect size also plays an important role and needs to be considered in drawing any statistical inference. The effect size in the functional connectivity between SM and SF in this study was about 0.5, which was larger than the effect sizes for remaining six findings that did not survive Bonferroni correction. It is evident from Figure 6 that the prevailing method of spatial normalization and smoothing reduces the effect size significantly. In fact, six of eight significant age-related DMN connectivity changes reported in Andrews-Hanna et al. (2007) have effect size smaller than 0.2. The large effect size in our significant findings on the right hemisphere can be considered as additional evidence that age-related disruption in resting-state BOLD fMRI functional connectivity is a unilateral phenomenon in the human brain.

The proposed native space method uses an fMRI localization algorithm which is based on gross morphological features of the brain; however, we should emphasize that functional units/nodes or cytoarchitecture in the brain do not necessarily match morphological features such as sulci and gyri. In addition, cytoarchitecture is highly variable between individuals. Thus, the proposed native space method should be considered as one step forward toward perfecting intersubject alignment, but by no mean will it completely remove all uncertainties. In addition, the existence of EPI distortion in fMRI image and our lack of reverse polarity acquisition to correct for it forced us to visually inspect the accuracy of intermodal registration in all 51 subjects in the data set. As the regional time series are obtained by averaging all voxels within a region, an inaccuracy of up to a voxel size was tolerated in this work without having a significant impact on the final results.

Analysis of the fMRI data in subjects' native space has a very high level of spatial correspondence accuracy in comparison with approaches that use spatial normalization into common space, but it is forced into a set of predefined regions motivated by the neuroanatomy of the human brain. Nonetheless, the method is extendable to any different set of brain regions. For this study, we used FreeSurfers' predefined cortical and subcortical regions. Localization accuracy of native space method directly depends on the accuracy of the underlying T1 image segmentation. Thus, any inaccuracy in the underlying T1 image segmentation will directly affect the localization accuracy in the native space. Even though FreeSurfer segmentation was reported to be comparable to manual segmentation (which is the silver standard in the field), an extra effort was made to check the accuracy of the underlying segmentation. We manually double-checked the FreeSurfer's results for any possible error, and corrections were made when needed.

By analyzing the resting-state BOLD fMRI data in subjects' native space, we achieved a higher between-subject localization accuracy which increased our statistical power to detect alterations in DMN connectivity in each hemisphere independently. Such advantages made the detection of significant unilateral disruption in the connectivity of DMN possible. The prevailing method of spatial normalization and smoothing failed to find such effect under the same conditions. In addition, the commonly accepted practice of interhemispheric averaging not only prevents analysis of two hemispheres independently, it also appeared to be a separate source of inaccuracy and seems to be problematic in practice. Our unilateral significant finding between supramarginal gyrus and superior-frontal cortex survived Bonferroni correction, had a large effect size, and correlated with cognitive performance. These observations support the hypothesis of unilateral disruption of DMN; however, replication of these findings with a larger number of samples is needed to further validate this hypothesis.
